# Design of AgNPs loaded γ-PGA chitosan conduits with superior antibacterial activity and nerve repair properties

**DOI:** 10.3389/fbioe.2025.1561330

**Published:** 2025-05-16

**Authors:** Yang Qu, Yinglei Ma, Heng An, Meng Zhang, Haoran Jiang, Bohan Xing, Bojiang Wang, Yanqun Liu, Yongqiang Wen, Peixun Zhang

**Affiliations:** ^1^ Department of Orthopedics and Trauma, Peking University People’s Hospital, Beijing, China; ^2^ Key Laboratory of Trauma and Neural Regeneration, Peking University, Beijing, China; ^3^ National Trauma Center, Peking University People’s Hospital, Beijing, China; ^4^ Beijing Laboratory of Trauma and Nerve Regeneration, Peking University, Beijing, China; ^5^ Peking University People’s Hospital Qingdao Hospital, Beijing, China; ^6^ School of Chemistry and Biological Engineering, University of Science and Technology, Beijing, China; ^7^ Peking University People’s Hospital Qingdao Hospital, Qingdao, China; ^8^ Department of Orthopedic Surgery, Yanbian University Hospital, Yanji, China

**Keywords:** γ-PGA, AgNPs, chitosan, antibacterial activity, peripheral nerve repair

## Abstract

To address the challenge of infections during peripheral nerve defect repair, this study introduces a γ-polyglutamic acid (γ-PGA) polymers designed to encapsulate silver nanoparticles (AgNPs). The AgNPs loaded γ-PGA polymers is applied as a coating on both the inner and outer surfaces of chitosan nerve conduits, providing antibacterial protection across the conduit. The antibacterial mechanism leverages the potent antimicrobial activity of nanosilver and the negatively charged field of γ-PGA, which repels bacterial adhesion to cell membranes. This dual mechanism significantly reduces the incidence of infection, which is a critical complication during nerve repair (Tavakolian et al., 2020). Furthermore, the pH-responsive dissociation behavior of γ-PGA allows for tunable antibacterial performance by modulating the pH environment. The composite nerve conduit demonstrates sufficient mechanical strength and hydrophilic properties, ensuring its stability and compatibility for implantation. *In vitro* antibacterial assays revealed outstanding antimicrobial performance, while biocompatibility evaluations confirmed an environment conducive to nerve cell proliferation and regeneration. This innovative nerve conduit material presents a promising solution for combating infections in nerve repair and regeneration. Its versatility and effectiveness suggest potential applications in complex neural repair scenarios, positioning it as a viable candidate for *in vivo* nerve regeneration therapies.

## 1 Introduction

Peripheral nerve injury (PNI) is a common clinical condition caused by various factors, including trauma, soft tissue compression, diabetes, and surgical procedures (Vijayavenkataraman, 2020). It affects over 1 million individuals annually, imposing a significant burden on quality of life and socio-economic systems. Severe cases of PNI can lead to loss of function, sensory abnormalities, or even limb amputation (Du et al., 2023). In clinical practice, polytrauma—particularly open multiple injuries—frequently involves peripheral nerve damage and deficits. Even with meticulous debridement, the local area of open wounds often carries a significant risk of infection. This heightened infection risk poses a substantial challenge to the effective repair of damaged nerves, severely impeding the regeneration process. To promote and achieve the repair of PNI, scientists have developed a range of implantable nerve repair materials through advancements in tissue engineering. However, the widespread application of these materials has been accompanied by an increasing risk of infection (Qian et al., 2022). Despite significant progress in infection prevention technologies and aseptic surgical practices, infection rates remain alarmingly high (Ye et al., 2023). Moreover, the overuse of antibiotics has exacerbated bacterial resistance, leading to the emergence of multidrug-resistant strains (Huang Y. et al., 2024). These challenges undermine the efficacy of traditional antimicrobial agents and infection-control measures, presenting an urgent need for innovative strategies to address infections in the context of nerve repair (Li S. et al., 2023; Septimus, 2018; Sun et al., 2023). Developing antibacterial nerve repair materials that balance biocompatibility and infection control is critical for advancing the treatment of PNI and improving patient outcomes.

To address the critical need for infection control during nerve defect and transection repair, numerous novel antibacterial materials have been developed (Zhang et al., 2024; Wang et al., 2024; Hou et al., 2023). However, materials with exceptional antibacterial efficacy often exhibit varying degrees of toxicity, which may hinder tissue repair and regeneration (Zhao et al., 2023; Shi et al., 2016). Among the few materials that combine excellent biocompatibility with antibacterial properties, most exhibit rapidly diminishing and uncontrollable antibacterial performance over time (Chen et al., 2023; Li et al., 2023b). This limitation compromises their long-term effectiveness in managing infections and supporting nerve regeneration. As a result, the regulation of antibacterial performance has emerged as a promising strategy to mitigate inflammation during nerve repair while facilitating tissue regeneration (Li et al., 2019). Developing materials that maintain a controlled, sustained antibacterial effect while supporting neural growth offers an ideal pathway for addressing the dual challenges of infection prevention and functional recovery in peripheral nerve repair.

Nano silver is characterized by its broad-spectrum antibacterial efficacy, strong performance, and high safety profile (Wang Z. et al., 2023). It exerts its antimicrobial effects through the release of silver ions (Ag^+^) and the induction of reactive oxygen species (ROS), mechanisms that have been extensively validated in research. Studies have confirmed the potent antibacterial activity of nano silver ions against *Escherichia coli* and *Staphylococcus aureus* (Wei et al., 2022). The antibacterial effectiveness of nano silver is dependent on both its concentration and duration of action. At sufficiently high concentrations, nano silver disrupts bacterial viability by inhibiting respiratory chain dehydrogenase activity and damaging the surface structure of bacterial cell membranes (Yu et al., 2022). These processes lead to bacterial destruction, achieving bactericidal and bacteriostatic effects. This makes nano silver a promising candidate for infection control in biomedical applications, particularly in environments requiring sustained and potent antibacterial action (He et al., 2023).

γ-Polyglutamic acid (γ-PGA), an amino acid polymer derived from microbial fermentation, exhibits remarkable properties such as high-water absorption, biodegradability, and excellent biocompatibility (Xu et al., 2024). These characteristics have made γ-PGA-based polymers widely applicable in the fields of biomedicine and tissue engineering (Cao et al., 2018). In addition to its intrinsic properties, γ-PGA carries a negative charge, which allows it to resist bacterial adhesion due to the similarly negative surface charge of most Gram-positive and Gram-negative bacteria. This characteristic is dependent on the dissociation of γ-PGA, which is influenced by pH levels (Wang et al., 2022). The antimicrobial capability of γ-PGA can therefore be modulated by adjusting the pH of the microenvironment, enabling control over its antibacterial performance. However, studies have shown that γ-PGA polymers also possess the ability to adsorb metal ions, enabling their combination with other components to form novel composite (Hu et al., 2023). These findings highlight the potential of γ-PGA as a multifunctional biomaterial. Its antibacterial surface properties, coupled with its capacity to incorporate metal ions, make it a promising candidate for developing advanced polymers tailored to specific biomedical applications (Yin et al., 2020).

**FIGURE 1:**
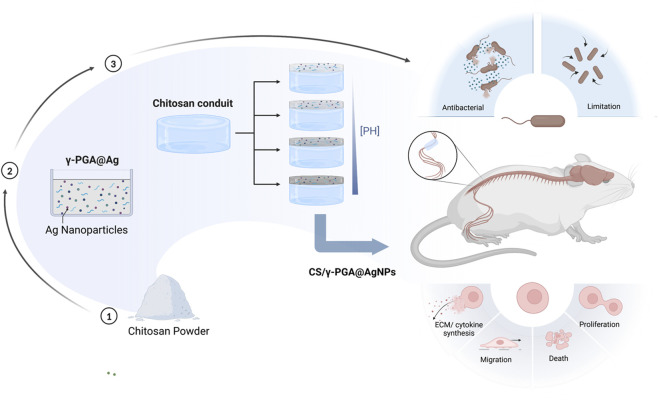
Schedule of the whole research, including the preparation of the CS/γ-PGA@AgNPs, the influence of PH in this composite, the antibacterial examination of the conduits, the evaluation of peripheral nerve repair effect.

To address the challenge of infection during the peripheral nerve repair process, our team built upon previous research on chitosan nerve repair conduits (Bu et al., 2023). By leveraging the unique properties of silver nanoparticles (AgNPs) and γ-polyglutamic acid (γ-PGA), we designed and fabricated a chitosan nerve repair conduit with a γ-PGA@AgNPs coating. This novel conduit aims to mitigate local infections during peripheral nerve repair. Comprehensive evaluations were conducted, including physicochemical characterization, biocompatibility assessment, antibacterial efficacy testing, and functional verification of its nerve repair capabilities (Figure 1).

In recent years, numerous antibacterial material systems have been developed to address infection control in peripheral nerve repair (Liu et al., 2022). For instance, researchers reported a graphitic carbon nitride-based antibacterial system, yet faced challenges in balancing biocompatibility with long-term antimicrobial efficacy (Egghe et al., 2023). Wang designed a gold-silver-carbon quantum dots hybrid composite with broad-spectrum antibacterial activity; however, its dependency on external photothermal stimulation limits applications in deep tissues (Permyakova et al., 2021). Additionally, starch-based antibacterial materials developed exhibited short-term efficacy due to their single-mode antimicrobial mechanism, failing to meet the prolonged demands of open trauma repair (Fischer et al., 2021). Although pH-responsive systems, such as the MOF-mediated AgNPs@ZIF-8 proposed by, enable environment-regulated antimicrobial release, issues like nanoparticle aggregation and mismatched release kinetics with nerve regeneration phases persist (Xie et al., 2023). In contrast, this study introduces a dual-functional chitosan conduit coated with γ-polyglutamic acid (γ-PGA) and silver nanoparticles (AgNPs) (CS/γ-PGA@AgNPs), which demonstrates three key innovations: (1) Synergistic dual antibacterial mechanisms: The negatively charged γ-PGA layer electrostatically repels bacterial adhesion, while AgNPs exert multi-target bactericidal effects, overcoming the limitations of single-mechanism materials (Huang Z. et al., 2024). (2) pH-responsive and controlled release: Leveraging the pH-dependent dissociation of γ-PGA, the conduit achieves efficient AgNPs release at infection sites (neutral pH ∼7.4) while minimizing release in healthy tissues, thereby balancing antibacterial potency and biosafety (Zhao et al., 2020). (3) Integrated functionality: The conduit maintains mechanical robustness (>1 N tensile strength), hydrophilicity (contact angle <30°), and tunable degradation (50% within 6 months) synchronized with nerve regeneration timelines, resolving the functional dichotomy between “antimicrobial action” and “tissue regeneration” observed in conventional materials (Rahmati et al., 2021). This design not only circumvents antibiotic resistance risks but also provides a novel strategy for infection control and functional recovery in complex traumatic environments.

## 2 Materials and results

### 2.1 Preparation and characterization of CS/γ-PGA@AgNPs conduits

The chitosan-based biological conduit fabrication protocol comprises the following steps: Initially, a 4% chitosan solution (degree of deacetylation >80%, viscosity 1,500 cP, Sigma-Aldrich) was prepared by dissolving chitosan in 2% acetic acid, followed by blending with a 1.6% gelatin solution (Sigma-Aldrich) to achieve a composite solution with a viscosity of 80,000–90,000 cP. The mixture was filtered through a 250-mesh stainless steel sieve and degassed for 24–48 h. The composite solution was extruded over Kirschner wires under controlled slow-speed drawing to form fine-diameter conduits, which were immediately immersed in 5% NaOH for structural fixation. The conduits were subsequently neutralized via deionized water rinsing (pH 7.0 verified by pH test strips) and soaked overnight. Dehydration was performed through sequential acetone immersion (2 min) and air-drying (10 min), followed by acetylation in a methanol/acetic anhydride (1:1 v/v) solution for 5–10 min until transparency was achieved. The final conduits were stored in 75% ethanol to prevent desiccation.

For γ-PGA/Ag coating deposition, acetylated conduits underwent surface pretreatment involving triple deionized water rinsing to remove residual reagents, followed by 0.1 M NaOH (37°C, 30 min) immersion to enhance surface hydroxyl group reactivity. An activation solution containing γ-PGA (0.1 g, MW = 700,000, Sigma-Aldrich), N-(3-dimethylaminopropyl)-N′-ethylcarbodiimide hydrochloride (EDC, 0.28 g, MW = 191.7, Macklin), and N-hydroxysuccinimide (NHS, 0.17 g, WM = 115.09, Macklin) in 30 mL deionized water (pH 4.8–5.2 adjusted with 0.1 M HCl) was stirred at 25°C for 2 h (Yu et al., 2023). The conduits were then immersed in the activated solution under nitrogen atmosphere and subjected to oscillatory grafting (37°C, 120 rpm, 5 cm amplitude) for 12 h. Sequential gradient silver loading was performed by immersion in 0.5%, 1.0%, and 1.5% AgNO3 solutions (light-protected, 37°C, 2 h each), followed by UV reduction (365 nm, 10 cm distance, 30 min). Post-reduction cleaning included triple deionized water rinsing, ethanol sonication (5 min), and ambient drying.

Silver nanoparticles (AgNPs) were synthesized by reducing silver nitrate (AgNO_3_, 1% w/v) with ascorbic acid (0.1 M, pH 7.0) under ambient conditions. The negatively charged carboxylate groups of γ-PGA electrostatically adsorbed Ag^+^ ions, acting as nucleation sites during reduction to prevent AgNPs aggregation. Covalent crosslinking between γ-PGA and chitosan conduits was achieved via EDC/NHS (molar ratio 1:1), immobilizing AgNPs within the polymer network (Lu et al., 2024). Therefore, to get better stability control, the reaction pH was maintained at 7.0 (phosphate buffer) to stabilize γ-PGA’s charge distribution and prevent AgNPs oxidation (Arafat et al., 2020). Ultrasonication (40 kHz, 15 min) ensured homogeneous dispersion of AgNPs within the γ-PGA matrix (Wang Y. et al., 2023). The γ-PGA/Ag-coated conduits were stored in sterile 75% ethanol under light-protected, sealed conditions.

Mechanical Properties: The tensile strength of the CS/γ-PGA@AgNPs conduits was evaluated using a universal tensile testing machine (MALK-10). Surface Morphology: The surface characteristics of the samples were analyzed using a scanning electron microscope (SEM, Hitachi SU-80). Hydrophilicity: Surface hydrophilicity was assessed using a static water contact angle measurement system (OCA 20, Dataphysics, Germany). Contact angles were measured at three random points on three identical samples, and the average value was calculated.

Ag^+^ Release Study: To investigate silver ion release kinetics, the samples were immersed in phosphate-buffered saline (PBS, 5 mL) and incubated at 37°C for 28 days. At predefined intervals, the supernatant was collected, and the released Ag^+^ concentration was quantified using inductively coupled plasma mass spectrometry (ICP-MS, PerkinElmer NexION 300XX). This comprehensive approach allows for the evaluation of the mechanical, morphological, hydrophilic, and release properties of the CS/γ-PGA@AgNPs conduits, providing critical insights into their potential for biomedical applications.

### 2.2 Antibacterial performance evaluation

Bacterial Models: *S. aureus* (ATCC 25923) and *E. coli* (ATCC 25923) were selected as model organisms for Gram-positive and Gram-negative bacteria, respectively. Sample Preparation: All samples were sterilized under UV light and placed in a 24-well plate. Bacterial Suspension: A bacterial suspension (1 mL, 1 × 10^7^ CFU/mL) was added to each well and incubated for 6 h.

Antibacterial Rate Measurement:After incubation, the samples were gently rinsed twice with PBS to remove unattached bacteria and transferred to new 5 mL test tubes. The adhered bacteria were detached using ultrasonication (40 W) for 5 min followed by 30 s of vortexing. The resulting solution was serially diluted and spread-plated onto agar plates in triplicates. The plates were incubated for 24 h, photographed, and the number of colony-forming units (CFU) was counted. The antibacterial rate (%) was calculated using the formula below:

$$\text{Antibacterial rate }\left(\%\right)=\left({\text{CFU}}_{\text{control}}\;{\text{CFU}}_{\text{experimental}}\right)/{\text{CFU}}_{\text{control}}\times 100\%.$$

Bacterial Viability Assay: Following co-culture with the samples, bacterial viability was assessed using the Alamar Blue assay (1:10 dilution, ThermoFisher, Catalog #DAL1100). The bacteria were incubated with the reagent in the culture medium for 4 h. Absorbance was measured at wavelengths of 540–590 nm using a microplate reader (SpectraMax, Molecular Devices). All measurements were performed in triplicate and repeated across three independent experiments to ensure reliability. This methodology provides a robust evaluation of both the antibacterial efficacy and the viability of bacteria in response to the tested materials.

### 2.3 *In Vitro* biocompatibility assessment

Live/Dead Cell Staining: All samples were rinsed three times with PBS to remove residual media. Staining Procedure: Samples were stained for 15 min in the dark using a combination of SYTO9 and propidium iodide (PI, ThermoFisher, Catalog #L7012). SYTO9 stains live cells (green fluorescence). PI stains dead cells (red fluorescence). Microscopic Observation: The stained samples were examined under a fluorescence microscope to differentiate live (green) and dead (red) cells. Measurements were conducted at three time points: Day 1, Day 3, and Day 7.

Cell Proliferation Assay: Co-Culture: Extract solutions from the samples were co-cultured with RSC96 cells. Proliferation Monitoring: The proliferation of RSC96 cells was assessed using the CCK-8 assay. The co-culture was incubated in a dark environment (wrapped in foil) for 3 h within a cell incubator. At intervals of 24, 48, and 72 h, absorbance at 450 nm was measured using a microplate reader.

Reproducibility: Each measurement was repeated three times for accuracy and statistical reliability. This dual approach—combining live/dead cell staining and cell proliferation analysis—provides a comprehensive evaluation of the material’s biocompatibility, ensuring both cell viability and growth are adequately supported.

### 2.4 Evaluation of nerve repair efficacy

Four-week-old female Sprague-Dawley (SD) rats were used as standard animal models for clinical research. The rats were divided into four groups based on the repair materials: sham surgery group (no nerve transection, n = 6), surgery group (nerve transection without repair, n = 6), chitosan nerve conduit group (CS, n = 8) and CS-γ-PGA@AgNPs nerve conduit group (n = 8). The sample size was determined based on power analysis (α = 0.05, β = 0.2) to ensure statistical validity, accounting for potential attrition. During the experiment, the sciatic nerve was carefully dissected and transected. For the chitosan nerve conduit group, prefabricated chitosan scaffolds were used for sutured repair, while the CS-γ-PGA@AgNPs group utilized nerve conduits coated with AgNPs and γ-PGA. All groups were monitored for 12 weeks post-surgery, aligning with the established timeline for peripheral nerve regeneration in rats. Twelve weeks post-surgery, the regenerated sciatic nerves were observed and assessed. Immunofluorescence staining (NF200, S100, and DAPI) was employed to evaluate the speed and quality of peripheral nerve regeneration. The analysis was performed using the ImageJ software, providing a quantitative measure of nerve regeneration outcomes.

### 2.5 *In vivo* functional assessment

In rats with peripheral nerve injury (PNI), histological nerve regeneration alone is insufficient for evaluating functional recovery. Therefore, postoperative muscle function recovery was assessed across all groups. The rats were dissected, and the soleus and gastrocnemius muscles were harvested for histological analysis. Tissue sections were stained with Masson’s trichrome and examined microscopically to evaluate muscle structure and density. ImageJ software was then used for subsequent image analysis to quantify muscle regeneration and assess the functional recovery facilitated by different nerve repair materials.

## 3 Results and discussion

### 3.1 Design and characterization of CS/γ-PGA@AgNPs conduits

A γ-PGA-based antibacterial polymers was synthesized using a room-temperature crosslinking method: γ-PGA was dissolved in deionized water under constant stirring. Silver Nanoparticle Formation: A silver nitrate (AgNO_3_) solution was added, followed by ascorbic acid to reduce silver ions into silver nanoparticles (AgNPs) (LI et al., 2023c). Crosslinking: EDC was introduced as a crosslinker to promote the formation of γ-PGA polymers. Purification: The reaction proceeded for 24 h, after which unreacted components were washed away with deionized water, yielding the γ-PGA-based antibacterial polymers.

**FIGURE 2:**
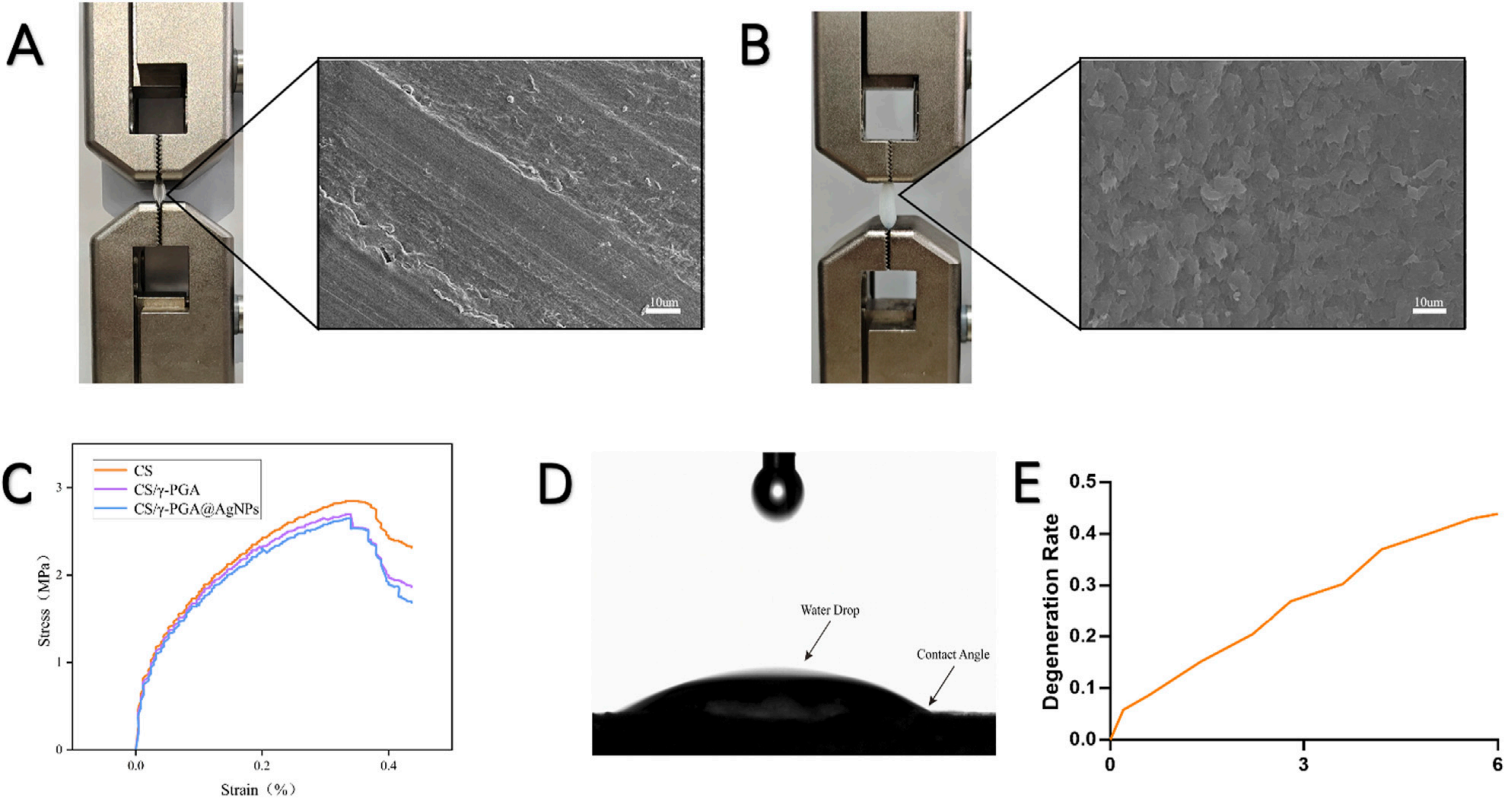
**(A)** Tensile test and scanning Electron Microscopy (SEM) images of CS neural conduit; **(B)** Tensile test and scanning SEM images of CS/γ-PGA@AgNPs conduit; **(C)** Stress-strain curve of CS/γ-PGA@AgNPs conduit, exhibiting a maximum tensile strength exceeding 1 N; **(D)** Contact angle measurement results of CS/γ-PGA@AgNPs conduit; **(E)** Degradation profile of CS/γ-PGA@AgNPs conduit over 6 months.

The γ-PGA polymers coated antibacterial conduits demonstrated excellent mechanical and tensile performance. Tensile testing was conducted using a universal tensile machine to evaluate the mechanical integrity of the synthesized conduits. Stretching Capacity: Conduits with an initial length of 18 mm could be stretched to a length of 29 mm, representing an elongation exceeding 150% of the original deformation. Stress-Strain Analysis: A stress-strain curve was generated after tensile testing (Figures 2A,B), revealing that the conduits could withstand tensile forces exceeding 1N (Figure 2C). The addition of the γ-PGA polymers coating did not compromise the structural integrity of the original nerve conduits. The coated conduits maintained excellent flexibility and mechanical strength, essential characteristics for surgical applications in peripheral nerve repair. The degradation profile of the CS/γ-PGA@AgNPs antibacterial conduit was evaluated over a 6-month period to determine its suitability for peripheral nerve repair applications. As shown in Figure 2E, the conduit exhibited gradual degradation over the course of the study. After 6 months, approximately 50% of the conduit was degraded, indicating a steady and controlled breakdown. This degradation timeline aligns well with the phased requirements of peripheral nerve repair, ensuring that the conduit provides structural and antibacterial support during the critical early phases of nerve regeneration, while gradually resorbing to allow for natural tissue remodeling (Yang et al., 2013). The degradation characteristics of the CS/γ-PGA@AgNPs conduit confirm its compatibility with the temporal demands of nerve healing, reinforcing its potential as an effective bioresorbable scaffold for clinical use (Contreras et al., 2023).

The hydrophilic properties of the CS/γ-PGA@AgNPs conduits were evaluated using a contact angle goniometer. The results of the contact angle measurements are shown in Figure 2D, indicating the hydrophilic nature of the polymers surface. Enhanced Antibacterial Efficiency: The hydrophilic surface facilitates the attraction, contact, and encapsulation of surrounding bacteria, ensuring close interaction with the silver nanoparticles embedded in the polymers. This promotes the polymers’s antibacterial efficacy (Ghaffar et al., 2024). Compatibility with Nerve Repair: The hydrophilic nature of the coating aligns with the requirements for nerve repair conduits, supporting cell attachment, nutrient exchange, and reducing bacterial adhesion (Filipović et al., 2020). This hydrophilic characteristic reinforces the dual functionality of the polymers as both an effective antibacterial agent and a supportive material for peripheral nerve regeneration.

### 3.2 Biocompatibility of CS/γ-PGA@AgNPs antibacterial conduits

Biocompatibility is a critical factor for polymers-based materials, as it directly impacts their effectiveness in tissue repair. To evaluate this, the γ-PGA@AgNPs coated antibacterial conduits were tested *in vitro*.

**FIGURE 3:**
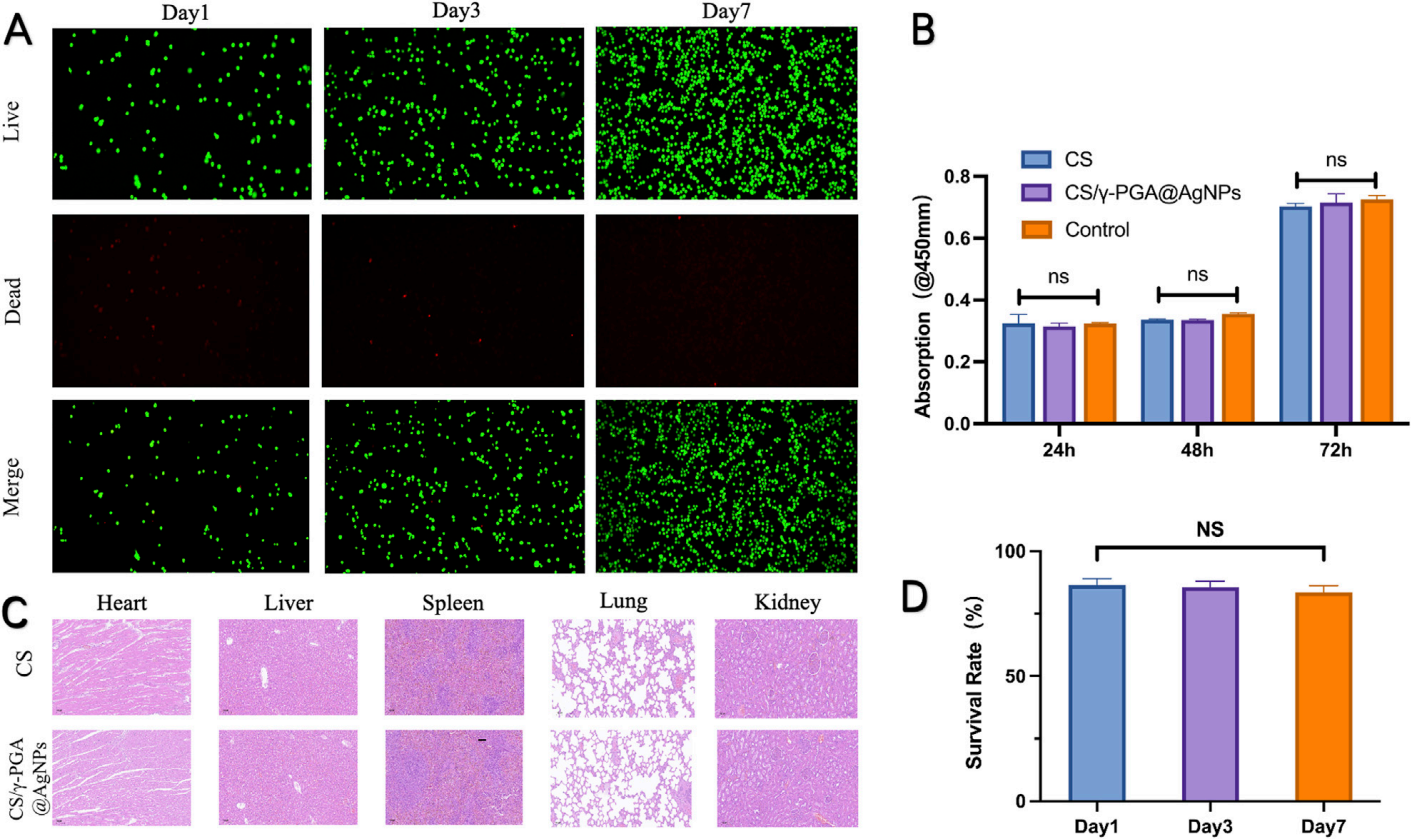
**(A)** Dead/Live Test of the CS/γ-PGA@AgNPs conduit in day1, day3 and day7; **(B)** the CCK-8 results of absorption in 450mm; **(C)** H&E stained section of heart, liver, spleen, lung and kidney; **(D)** Survival rate of nerve cells in day1, day3 and day7.

The antibacterial conduits were immersed in EMEM complete medium for 24 h to prepare the extract solution. Medium from conduits without coating served as the control group. CCK-8 Assay: The extract solutions were incubated with RSC96 cells, and cell viability was assessed using the CCK-8 kit. As shown in Figure 3B, there was no statistically significant difference in OD values between the experimental group (antibacterial conduits) and the control group (uncoated conduits). Live/Dead Cell Staining: Cells cultured with the extract were stained to assess viability. Over time, cell density increased without noticeable cell death in either group (Figure 3A). Statistical analysis confirmed no significant difference in cell growth between the experimental and control groups (Figure 3D). The *in vitro* studies confirmed that the CS/γ-PGA@AgNPs antibacterial conduits exhibit excellent biocompatibility. This ensures that the material supports cellular viability and proliferation without inducing cytotoxicity, making it suitable for use in peripheral nerve repair and regeneration (Qin et al., 2023).

To further confirm the biocompatibility of antibacterial nerve conduits, an *in vivo* subacute and chronic toxicity study was conducted (Cao et al., 2021). This experiment assessed the impact of the conduits on major organ systems in rats. Experimental and control groups of rats were utilized. Organ Analysis: major organs, including the heart, liver, spleen, lungs, and kidneys, were harvested from the test subjects. Tissue samples were processed into sections and stained with hematoxylin and eosin (H&E) for histopathological examination (Figure 3C). Morphological Assessment showed that no significant abnormalities in cellular morphology or tissue structure were observed in the experimental group compared to the control group. In addition, no evidence of tissue degeneration or widespread cell death was detected. Therefore, *in vivo* toxicity study demonstrated that γ-PGA @AgNPs antibacterial conduits exhibit excellent biocompatibility, with no adverse effects on the tested organ systems (Bertsch et al., 2023). These findings indicate that the implantation of the CS/γ-PGA@AgNPs conduit caused no discernible damage to internal organs. This *in vivo* experiment further substantiates the excellent biocompatibility of the γ-PGA antibacterial nerve conduit (Yan et al., 2022).

### 3.3 Antibacterial performance of CS/γ-PGA@AgNPs conduits

**FIGURE 4:**
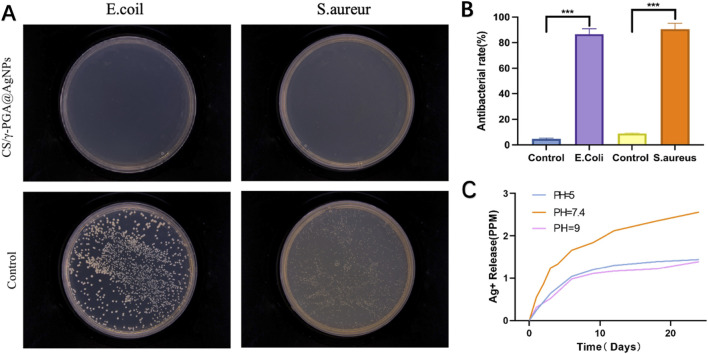
**(A)** Bacterial coating experiment results for *Escherichia coli* and S. aureur using CS/γ-PGA@AgNPs conduit; **(B)** Quantitative results of antibacterial Efficacy testing; **(C)** Release levels of AgNPs under different pH conditions.

The antibacterial efficacy of materials is crucial for postoperative recovery in peripheral nerve repair. This study evaluated the antibacterial properties of the γ-PGA@AGNPs conduits and their role in infection control during nerve regeneration. Chitosan, a key component of the nerve conduits, is well-documented for promoting axonal growth and enhancing neuronal function (Aghbashlo et al., 2023; Gholap et al., 2024). Its polycationic structure also imparts inherent antibacterial activity. Prior studies report chitosan conduits exhibit 30%–50% inhibition against *S. aureus* and 40%–60% inhibition against *E. coli*. Mixed suspensions of *S. aureus* (Gram-positive) and *E. coli* (Gram-negative) were used. Extracts from uncoated chitosan conduits and CS/γ-PGA@AgNPs antibacterial conduits were prepared. Bacterial suspensions were plated on culture media infused with the extracts. Incubation conditions: pH 7.3, 37°C, 12 h. Colony distribution was observed and quantified (Figures 4A,B). The results showed that CS/γ-PGA@AgNPs antibacterial conduits demonstrated >90% antibacterial efficiency against both *S. aureus* and *E. coli*. Statistical analysis confirmed significantly superior antibacterial performance compared to uncoated chitosan conduits. So, the γ-PGA@AGNPs coating significantly enhances the antibacterial properties of the nerve conduits, achieving infection control levels that exceed the requirements for peripheral nerve repair (Liang et al., 2023; Huang et al., 2022). This suggests its potential to effectively prevent postoperative infections and improve surgical outcomes in clinical applications.

Additionally,The antibacterial efficacy of the CS/γ-PGA@AgNPs conduit is attributed to two main mechanisms: negative Charges Generated by γ-PGA Dissociation; the dissociation of γ-PGA produces negative charges, which can disrupt bacterial membranes and inhibit bacterial adhesion. Both Gram-positive and Gram-negative bacterial membranes carry net negative surface charges. γ-PGA, a polyanionic polymer with densely distributed carboxyl groups, generates a strong negative surface charge. This charge similarity induces electrostatic repulsion between γ-PGA and bacterial membranes, preventing bacterial attachment to the conduit surface (Figure 4A). The conduits’s dissociation continuously releases AgNPs, which directly target and disrupt bacterial cell walls, enhancing the antibacterial effect.

At the molecular level, the carboxylate groups (-COO^-^) of γ-PGA create a high-density negative charge field (Viola and Mass, 2025). This field repels negatively charged components of bacterial membranes, effectively increasing the energy barrier for bacterial adhesion. The repulsive forces dominate at short distances (<10 nm), as described by Derjaguin-Landau-Verwey-Overbeek (DLVO) theory, thereby preventing bacterial colonization (Ohshima, 2024).

Since the dissociation of γ-PGA is pH-dependent, the release efficiency of AgNPs was evaluated under different pH conditions: acidic (pH = 5), neutral (pH ∼7.4, close to physiological conditions) and alkaline (pH = 9). The results (Figure 4C) showed that at neutral pH, the dissociation rate of γ-PGA was found to be the highest, leading to the fastest release of AgNPs. Both acidic and alkaline environments exhibited slower γ-PGA dissociation and reduced AgNPs release rates. To investigate the release kinetics, we fitted the experimental data to Korsmeyer-Peppas model. The experimental data yielded n = 0.48 (R2 = 0.96), indicating a non-Fickian (anomalous) diffusion mechanism. This suggests that Ag^+^ release is governed by a combination of polymer relaxation (swelling of the γ-PGA matrix) and Ag^+^ diffusion through the hydrated network. The findings suggest that the CS/γ-PGA@AgNPs conduit is optimally designed to exhibit enhanced antibacterial performance in physiological pH conditions, making it highly effective in mitigating bacterial infections during peripheral nerve repair *in vivo*. This characteristic highlights its potential as an advanced solution for clinical applications.

### 3.4 Evaluation of peripheral nerve repair effects of CS/γ-PGA@AgNPs antibacterial conduits in vivo

**FIGURE 5:**
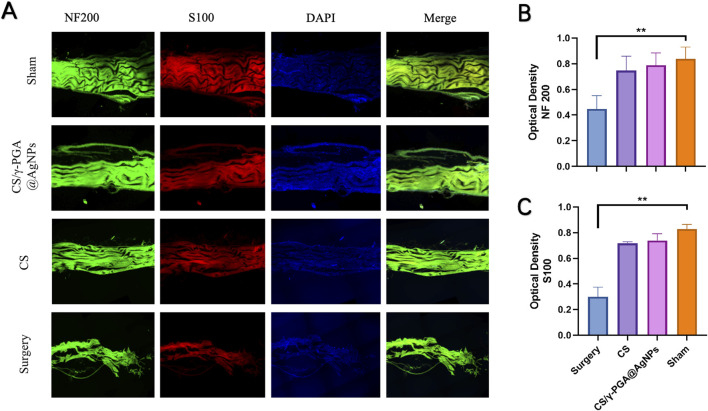
**(A)** Confocal fluorescence micrographs of immunostained cells in four groups: sham, CS/γ-PGA@AgNPs, CS and surgery; **(B)** Quantitative analysis of NF200 immunofluorescence staining; **(C)** Quantitative analysis of S100 immunofluorescence staining.

The transected nerve stumps exhibited varying degrees of healing across groups. Immunofluorescent staining with NF200 and S100, followed by confocal microscopy imaging, revealed the results shown in Figure 5A. Both the NF200 and S100 fields demonstrated that the sham surgery group exhibited the highest density of regenerated axonal fibers. In contrast, the chitosan nerve conduit group and the CS/γ-PGA@AgNPs implantation group displayed similar densities of regenerated axonal fibers, both significantly higher than those observed in the surgery-only group.

Quantitative analyses presented in Figures 5B,C further corroborate these findings. These results suggest that nerve conduits accelerate axonal regeneration. Furthermore, the inclusion of the antibacterial CS/γ-PGA@AgNPs coating not only retained the chitosan conduit’s regenerative benefits but also provided a low-infection-risk microenvironment conducive to Schwann cell regeneration. This facilitated the extension and regeneration of peripheral nerves to an even greater extent.

### 3.5 *In Vivo* functional assessment of target muscle recovery with CS/γ-PGA@AgNPs antibacterial conduits

**FIGURE 6:**
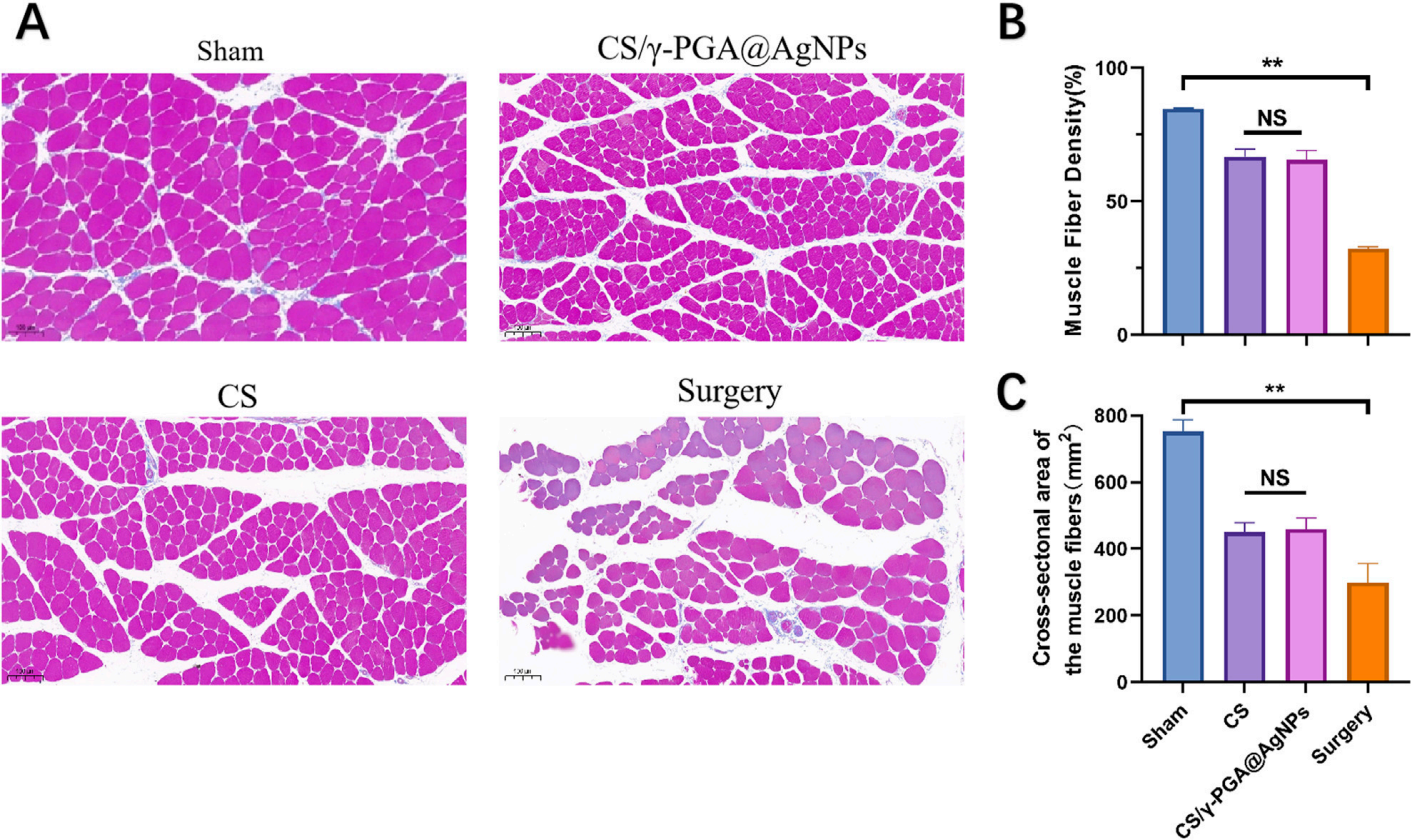
**(A)** Histological analysis of muscle sections: sham, CS/γ-PGA@AgNPs, CS and surgery; **(B)** Quantitative analysis of muscle fiber density between the above groups; **(C)** Quantitative analysis of Cross-sectional area of the muscle fibers between the above groups.

To evaluate the efficacy of γ-PGA@AgNPs antibacterial nerve conduits (CS/γ-PGA@AgNPs) in nerve repair, female Sprague-Dawley (SD) rats (4 weeks old) were selected as the standard animal model. Rats were divided into four groups based on surgical procedures and implanted materials: Sham Surgery Group: Sciatic nerve exposed without transection. Surgical Group: Sciatic nerve exposed and transected without repair. Chitosan Conduit Group: Sciatic nerve repaired using a prefabricated chitosan nerve conduit. CS/γ-PGA@AgNPs Group: Sciatic nerve repaired using the CS/γ-PGA@AgNPs antibacterial nerve conduit. All groups were monitored for 12 weeks post-surgery. Target muscles were harvested, sectioned, and stained with Masson’s trichrome. Muscle density was quantified and compared among groups (Figures 6B,C). The results showed that the sham surgery group exhibited the highest muscle density, indicative of intact nerve and muscle function. The surgical group displayed the lowest muscle density, reflecting the absence of nerve repair. Both the chitosan conduit group and the CS/γ-PGA@AgNPs group demonstrated improved muscle density compared to the surgical group (Figure 6A). The findings indicate that both CS/γ-PGA@AgNPs antibacterial nerve cond uits and chitosan nerve conduits significantly promote the recovery of neuromuscular function after surgery. Importantly, the implantation of CS/γ-PGA@AgNPs did not compromise the nerve repair-promoting effects inherent to chitosan nerve conduits. This underscores the dual benefits of the CS/γ-PGA@AgNPs antibacterial nerve conduit in effectively supporting nerve regeneration while simultaneously addressing postoperative infection risks.

## 4 Conclusion

In this study, we developed a multifunctional chitosan-based nerve conduit coated with γ-Polyglutamic acid (γ-PGA) and silver nanoparticles (AgNPs) to address the dual challenges of infection control and nerve regeneration in peripheral nerve repair. The γ-PGA@AgNPs coating exhibits a pH-responsive antibacterial mechanism, leveraging the electrostatic repulsion of γ-PGA’s negatively charged carboxyl groups and the sustained release of Ag^+^ ions via a non-Fickian diffusion process. This dual-action strategy achieved >90% antibacterial efficacy against both *S. aureus* and *E. coli* under physiological conditions (pH 7.4), while maintaining excellent biocompatibility, as evidenced by statistically validated cell viability assays and the absence of organ toxicity *in vivo*.

The conduit’s mechanical resilience (tensile strength >1 N, elongation >150%) and controlled degradation profile (50% mass loss over 6 months) ensure structural support during the critical phases of nerve regeneration. Its hydrophilic surface (contact angle <90°) further facilitates nutrient exchange and reduces bacterial adhesion, synergizing with the pH-triggered Ag^+^ release to create a low-infection-risk microenvironment. Notably, the material’s swelling behavior aligns with clinical requirements, balancing hydration dynamics and mechanical stability.

While this study focused on functional validation, future work will explore advanced characterization techniques, such as synchrotron X-ray tomography, to resolve dynamic swelling and degradation mechanisms. Additionally, integrating neurotrophic factors (e.g., NGF) into the γ-PGA matrix could further accelerate axonal regeneration. These innovations position the CS/γ-PGA@AgNPs conduit as a promising candidate for translational applications in complex neural repair scenarios, offering a robust alternative to traditional antibiotic-dependent therapies.

## Data Availability

The raw data supporting the conclusions of this article will be made available by the authors, without undue reservation.
