# Supplementary figures and images for: Design of AgNPs loaded γ-PGA chitosan conduits with superior antibacterial activity and nerve repair properties
Source: Front Bioeng Biotechnol. 2025 May 16;13:1561330. doi: 10.3389/fbioe.2025.1561330 (PMC12122499; doi:10.3389/fbioe.2025.1561330)

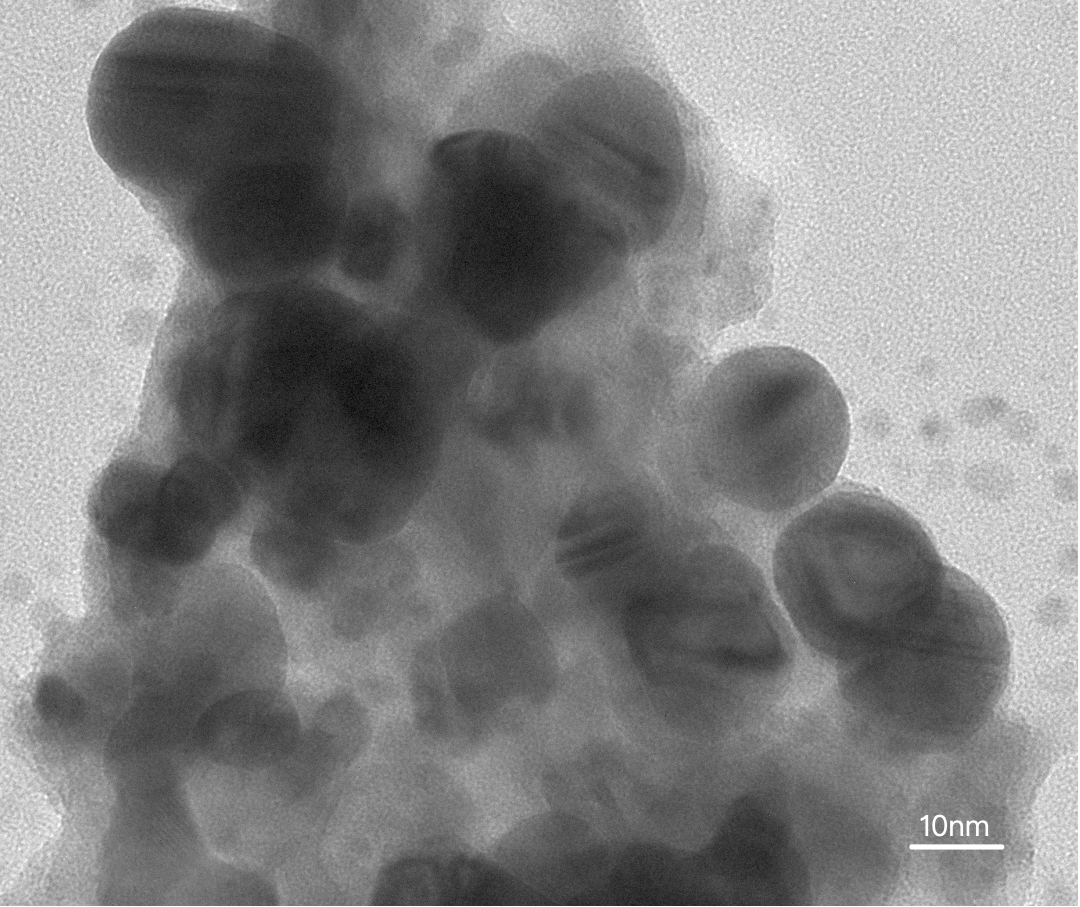

Supplement: Supplementary file 1 [file Image3.tif]

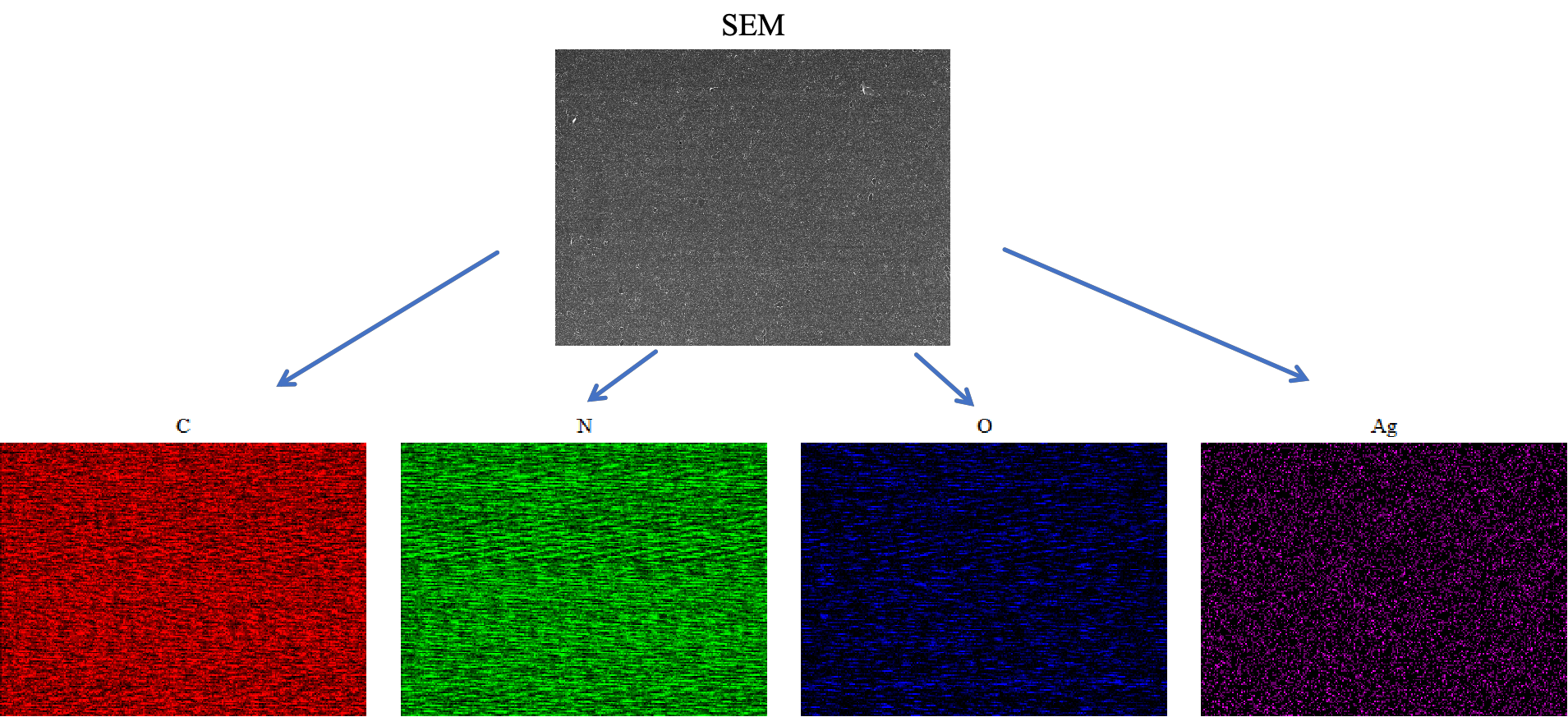

Supplement: Supplementary file 2 [file Image4.tif]

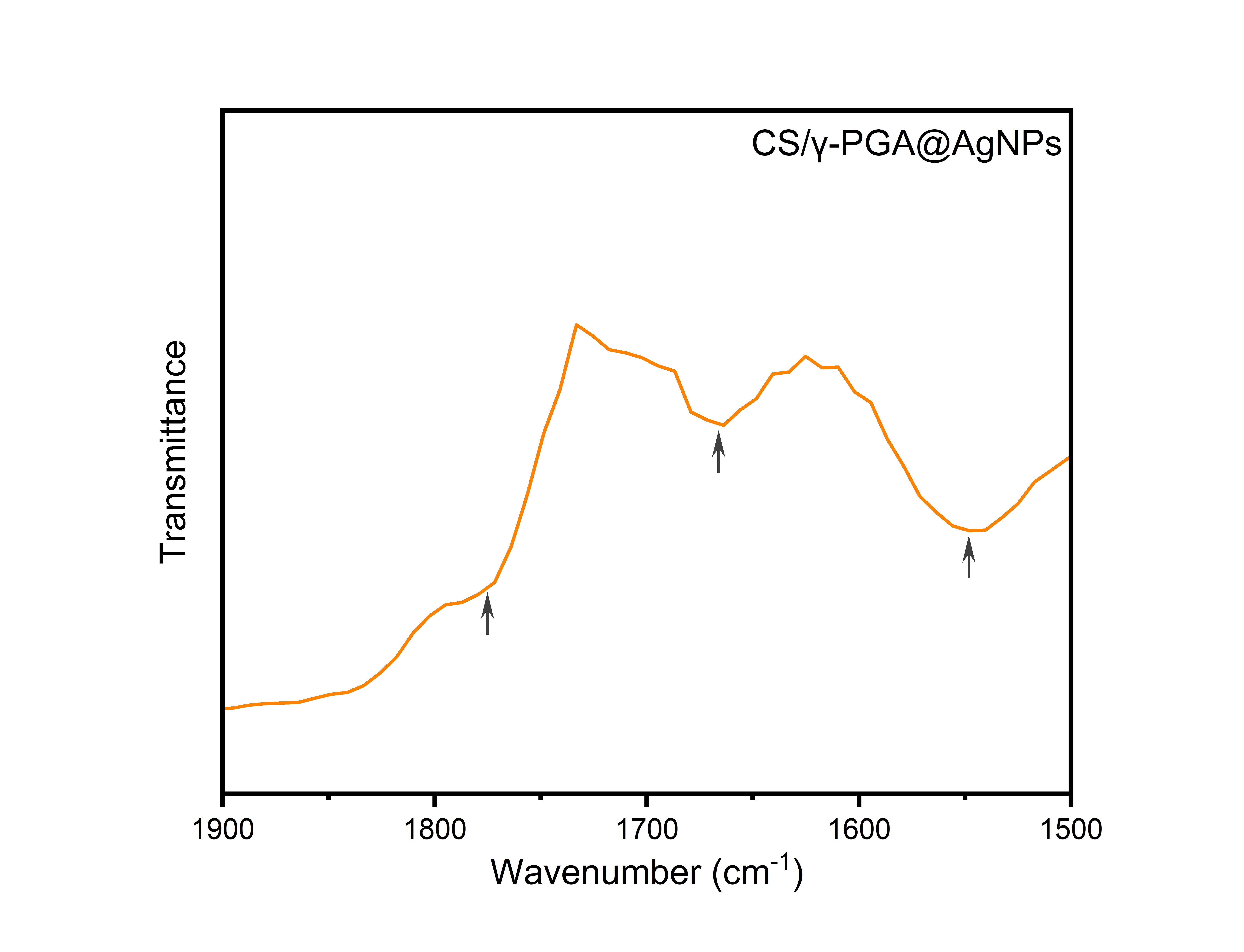

Supplement: Supplementary file 3 [file Image5.jpeg]

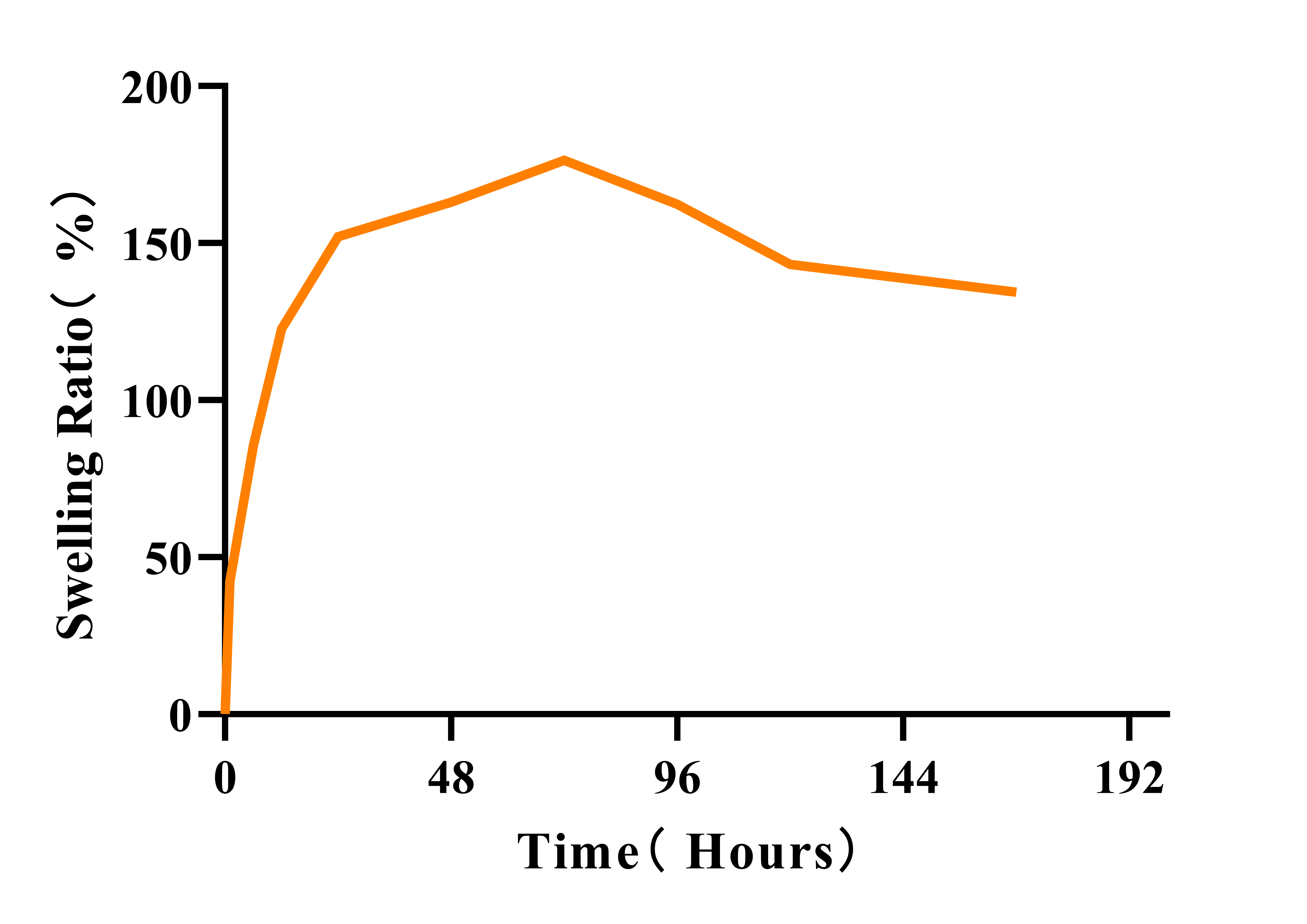

Supplement: Supplementary file 4 [file Image1.tif]

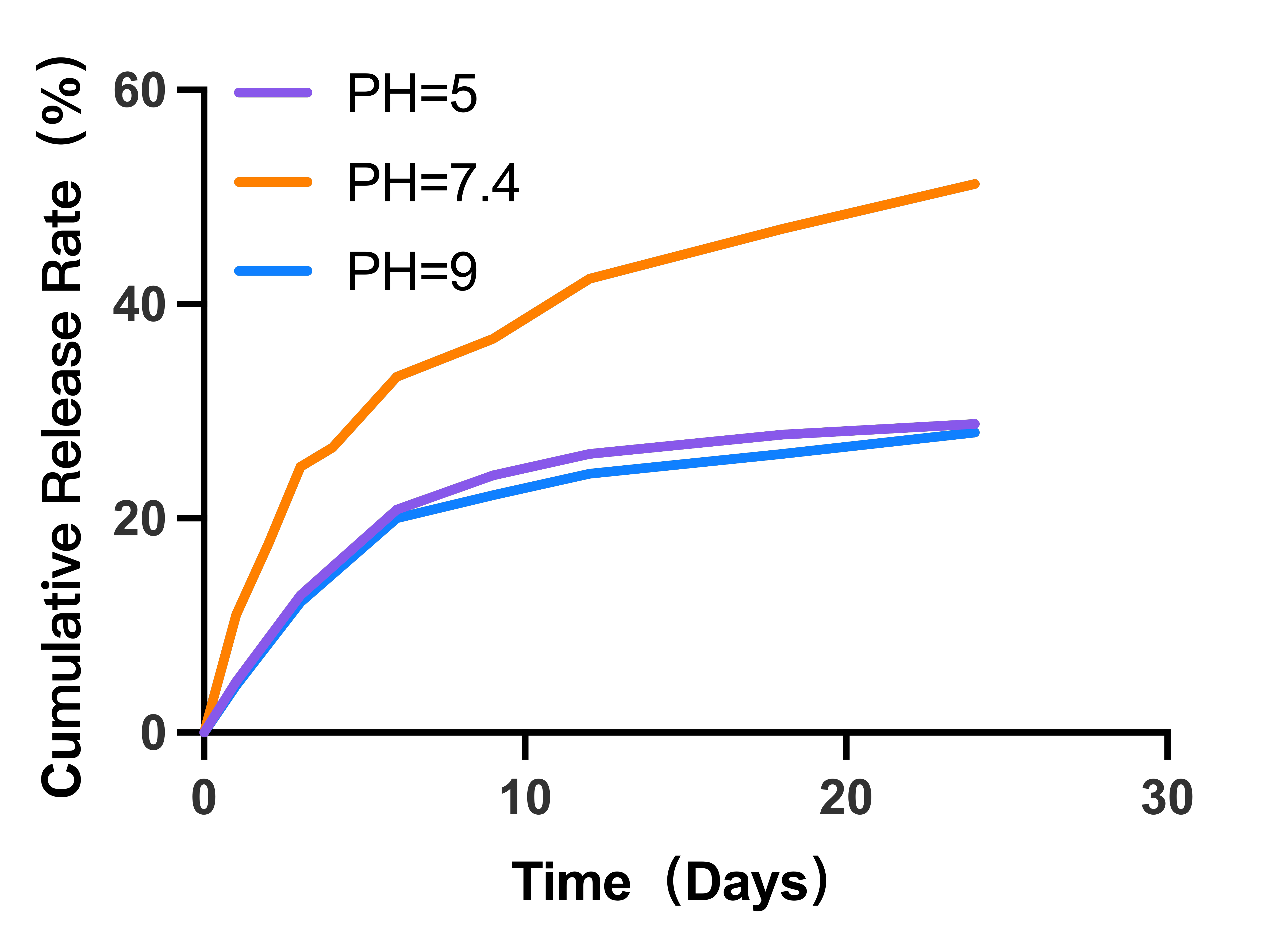

Supplement: Supplementary file 5 [file Image2.tiff]
